# Impact of an HIV Care Coordination Program on All-cause, HIV-related, and Non-HIV-related Mortality in Younger and Older Adults With HIV in New York City, 2018–2022

**DOI:** 10.1093/ofid/ofag206

**Published:** 2026-04-23

**Authors:** McKaylee M Robertson, Tigran Avoundjian, Faisal Abdelqader, Sarah L Braunstein, Denis Nash, Mary K Irvine

**Affiliations:** Institute for Implementation Science in Population Health (ISPH), City University of NewYork (CUNY), New York City, New York, USA; Graduate School of Public Health and Health Policy, City University of NewYork (CUNY), New York City, New York, USA; Bureau of Hepatitis, HIV, and Sexually Transmitted Infections, NewYork City Department of Health and Mental Hygiene, New York City, New York, USA; Bureau of Hepatitis, HIV, and Sexually Transmitted Infections, NewYork City Department of Health and Mental Hygiene, New York City, New York, USA; Bureau of Hepatitis, HIV, and Sexually Transmitted Infections, NewYork City Department of Health and Mental Hygiene, New York City, New York, USA; Institute for Implementation Science in Population Health (ISPH), City University of NewYork (CUNY), New York City, New York, USA; Graduate School of Public Health and Health Policy, City University of NewYork (CUNY), New York City, New York, USA; Bureau of Hepatitis, HIV, and Sexually Transmitted Infections, NewYork City Department of Health and Mental Hygiene, New York City, New York, USA

**Keywords:** care coordination, durable viral suppression, mortality, people aging with HIV, viral load suppression

## Abstract

**Background:**

Evidence-based strategies are needed to address the increasing healthcare system demands for people aging with HIV (PAWH). HIV case management models integrating access to primary care, specialty care, and supportive services represent a promising approach for healthy aging.

**Methods:**

We emulated a target trial comparing mortality outcomes through 36-month follow-up among people with HIV (PWH) enrolled in the New York City revised Care Coordination Program (CCR) versus a contemporaneous group of PWH who met CCR eligibility criteria but did not enroll in the CCR. To estimate the effect of the CCR on all-cause and cause-specific mortality, we used hazards regression with inverse probability of treatment weights controlling for clinical and demographic differences.

**Results:**

From August 2018 to March 2021, 3040 individuals enrolled in the CCR. At enrollment, 43% of CCR enrollees were aged 50 years or older, 27% had a CD4 count <200 cells/µL, and 19% were diagnosed prior to 1996. In adjusted analyses, CCR enrollees had lower all-cause mortality (adjusted hazard ratio [aHR]: 0.71 [0.60–0.84]), driven primarily by reductions in HIV-related deaths. The effects on all-cause and HIV-related mortality were consistent among PAWH and younger PWH, among those lacking evidence of viral suppression (VS), and among those with lower CD4 counts (<350 cells/µL).

**Conclusions:**

The CCR appears to reduce all-cause and HIV-related mortality including among PWH at highest risk for poor outcomes. These findings suggest that integrated care coordination models—linking primary care, specialty care, and psychosocial services—offer survival benefits and multiple health outcomes improvements among PWH.

## Teaser

Relative to usual care, the revised Care Coordination Program shows evidence of reducing all-cause and HIV-related mortality among populations at risk for poor outcomes, including people aging with HIV, younger people with HIV (PWH), and PWH lacking evidence of viral suppression.

The life expectancy of people with HIV (PWH) has dramatically increased due to improvements in HIV care and availability of antiretroviral treatment (ART) [1, 2]. Early treatment and improved survival on ART has led to an increased number of people aging with HIV (PAWH), generally defined as those aged 50 years or older [3, 4]. As the median age of PWH has increased, the promotion of healthy aging has become a priority of HIV care [5]. Among the 1.1 million PWH in the United States, the number of people aged 50 years or older is predicted to increase from 54% at the end of 2021 to 63%–75% by 2040 [6]. Risk of major contributors to mortality such as chronic illness is greater among PWH than people without HIV [7]. Evidence-based strategies are needed to address the growing complexity of caring for PAWH [8, 9].

Care coordination or other models integrating access to primary and specialty care, and including psychosocial and supportive services for PWH, represent one approach to promoting longevity. The New York City (NYC) Ryan White HIV/AIDS Program Part A (RWPA) HIV Care Coordination Program (CCP) combines multiple evidence-based strategies that aim to meet the medical and social service needs of clients: HIV case management by interdisciplinary teams, patient navigation, and health promotion and coaching around HIV self-management skills [10, 11]. In observational effectiveness analyses, the CCP improved short and long-term viral suppression (VS) relative to usual care for those consistently unsuppressed [12, 13]. On the basis of CCP effectiveness findings, the Centers for Disease Control and Prevention designated the CCP as an evidence-based intervention for linkage, retention, and re-engagement [14] and evidence-based structural intervention for VS [15, 16]. However, the CCP has not been evaluated for impact on mortality [12, 13, 16–18]. Furthermore, the broader evidence base on case management effectiveness has focused on more proximal outcomes (care engagement, adherence, and VS) than mortality.

In 2018, the NYC Health Department implemented a revised Care Coordination Program (CCR) to strengthen the intervention and reduce barriers to its delivery. The objective of our analysis was to examine the impact of the CCR on all-cause and HIV-specific mortality and among all persons enrolled and specific populations at risk for mortality, including PAWH.

## METHODS

### Care Coordination Intervention

The NYC Department of Health and Mental Hygiene (NYC Health Department) launched a multicomponent CCP in December 2009, using federal RWPA funding [17]. The CCP model implemented in 2009 combined several best practices, including a “medical home” approach; multidisciplinary care team communication; shared decision-making through case conferences; patient navigation, including accompaniment to primary care visits; ART adherence support; and a structured health promotion curriculum [17, 19, 20]. Based on effectiveness findings [12, 13, 16] and identified barriers, the NYC Health Department and their community partner on RWPA, the HIV Health and Human Services Planning Council of New York, outlined a redesign of the CCP in 2017–2018. The revised program (CCR) also aimed to meet the medical and social service needs of clients through interdisciplinary case management, patient navigation, health education, and promotion of HIV self-management skills, but incorporated features intended to increase program reach, flexibility, feasibility, and sustainability. The modifications included adding resources for HIV self-management; supporting videoconferencing options for directly observed therapy; replacing per-member-per-day reimbursement with a fee-for-service reimbursement model; emphasizing identification and recruitment of individuals with documented clinical need (eg, unsuppressed viral load [VL]); and removing enrollment tracks that prescribed fixed visit frequencies based on assessed client need, in favor of greater flexibility for client-centered care [11]. Intervention details have been published [10, 11].

CCR enrollment was open to PWH who were 18+ or emancipated minors, eligible for local RWPA services (based on residence within the New York eligible metropolitan area and household income ≤435% of federal poverty level [FPL] for enrollments through February 2019 and ≤500% of FPL for enrollments after February 2019), and demonstrating need for care coordination (based on recent HIV diagnosis, care or treatment initiation, absences from care, unsuppressed VL, hepatitis-C coinfection, pregnancy, ART regimen change, missed appointments, or provider-assessed risk of care interruption or treatment failure).

### Data Sources and Population

We merged longitudinal, population-based surveillance and programmatic data sources. Revised Care Coordination Program programmatic data were drawn from the NYC Health Department's Electronic System for HIV/AIDS Reporting and Evaluation (eSHARE). Ryan White HIV/AIDS Program Part A service providers have been contractually required to submit programmatic data through eSHARE since March 2011. Using eSHARE data, we identified all PWH enrolled in the CCR at any point from 01 August 2018, to 31 March 2021.

The HIV Surveillance Registry contains demographic (sex, race/ethnicity, and age at diagnosis) and transmission category information on all diagnoses of HIV reported in NYC, as well as comprehensive HIV-related laboratory reporting (including all CD4 and VL dates and results for tests ordered by NYC providers). Vital status information, including cause of death, is updated through regular matches with local and national death data to ascertain deaths occurring in and outside of NYC. For mortality outcomes, we used Registry data reported through 31 March 2024, which allowed for complete cause of death reporting through 31 March 2022.

### Research Ethics

The Institutional Review Board at the NYC Health Department approved this study (Protocol 18-009). For these secondary analyses of de-identified data, we received a waiver of informed consent under 45 CFR 46.116(d)(2).

### Target Trial Specification and Emulation

This observational analysis emulated a hypothetical randomized experiment, the target trial, to determine whether the CCR reduced mortality relative to usual care [21]. We first specified the protocol of a target trial to estimate the effect of the intervention on mortality. The hypothetical target trial protocol is summarized in Supplementary Table 1. Next, we emulated the design and intention-to-treat (ITT) analysis of the target trial.

### Contemporaneous Comparison Population

To assess the impact of the CCR on mortality, we identified a contemporaneous registry-based control group of PWH who met eligibility criteria for enrollment into the CCR but did not enroll in the CCR. We identified controls in a 3-step process [22]. First, we identified eligible persons and their eligibility windows, or ranges of time from 1 August 2018, to 31 March 2021, when an individual met eligibility criteria for the CCR. Registry-based eligibility criteria are defined in Supplementary Table 2.

Second, because we expect CCR effects to vary based on HIV treatment history, we ascertained baseline treatment status prior to each month of eligibility. The 4 baseline treatment status groups were defined in terms of diagnosis or VS in the 12 months prior to eligibility: (1) newly diagnosed, (2) consistently suppressed (≥2 VLs ≥90 days apart and all VLs ≤200 copies/mL), (3) lacking evidence of VS (all VLs reported >200 copies/mL or no VL report), or (4) inconsistently suppressed (≥1 VL ≤200 copies/mL, but not all VLs ≤200 copies/mL).

Finally, for each CCR enrollee within each baseline treatment status group, we randomly sampled 1 control from among those identified as eligible . We matched the distribution of starts of follow-up for eligible yet unenrolled controls with the distribution of enrollment dates (month and year) in the CCR. In this way, each selected control was assigned a pseudo-enrollment date based on a month and year in which they were eligible for CCR enrollment.

### Measures and Variable Definitions

#### Outcomes

We examined (1) all-cause, (2) HIV-related, and (3) all non-HIV-related deaths. We also examined the 2 most frequently occurring non-HIV-related causes: (3a) major cardiovascular disease and (3b) malignant neoplasms. We classified as “HIV-related” deaths for which the underlying cause recorded in the death certificate was coded with one of the International Classification of Diseases Tenth Revision (ICD-10) codes representing HIV disease, B20-B24, O98.8 or R75 [23, 24]. These codes correspond with opportunistic infections (eg, pneumocystis carinii pneumonia and cytomegaloviral infection), malignant neoplasms (Kaposi sarcoma and Burkitt lymphoma), other specific diseases or infections associated with HIV infection, and unspecified conditions consistent with HIV disease or AIDS. We classified as “non-HIV-related” all underlying causes that did not represent HIV-related causes. Major cardiovascular disease includes codes representing I00–I78 and non-HIV-related malignant neoplasms representing C00–C97 [25].

#### Covariates

We used the Registry to ascertain age, gender, race/ethnicity, HIV transmission category, VL, CD4 count, HIV diagnosis year, and baseline HIV treatment status. For VL and CD4 count, we used the most recent measure in the year before enrollment/pseudo-enrollment. Age 50+ has been proposed as the cut-point for defining PAWH, in recognition that PWH 50+ have poorer immune recovery after starting ART and greater burden of comorbidities relative to younger PWH [3, 4]. HIV diagnosis year was categorized in relation to HIV treatment eras with diagnoses <1996 representing the pre-combination ART (cART) era, 1996–2005 representing the cART era, and 2006 and later representing the single-tablet era.

Residential ZIP-code-level HIV prevalence and poverty were assigned based on each participant's most recent available ZIP code of residence in the Registry prior to enrollment/pseudo-enrollment. ZIP-code-level poverty data were obtained from the American Community Survey.

For CCR participants, we used eSHARE data to ascertain insurance status, housing status, recent drug use, mental health (reported diagnosis of depression/anxiety, psychosis, or posttraumatic stress disorder [PTSD]), and status. These variables were chosen because they were collected by CCR providers in the intake assessment and therefore could be used to identify clients in need of closer follow-up.

### Statistical Analysis

We used an ITI approach with stabilized inverse probability of treatment weights (IPTWs) to examine the effects of the CCR on mortality outcomes [26]. For the IPTW, we fit logistic regression models with CCR enrollment as the outcome for each baseline treatment status group, and we a priori–selected confounders as independent variables (Supplementary Table 3). We generated hazard ratios to estimate the effect of the CCR on all-cause and HIV-specific mortality. Individuals contributed time from enrollment to the earlier of 31 March 2022, 36 months (1095 days) postenrollment/pseudo-enrollment, or death. We looked at overall CCR effectiveness and effectiveness within subgroups based on treatment status, age (<50 and ≥50), race/ethnicity, diagnosis year, and baseline CD4 count. To estimate the effect of the CCR on mortality, we applied the stabilized IPTW to separate models for each outcome with a term for CCR enrollment, the covariate of interest, and an interaction term.

To assess the robustness of the results to unmeasured or uncontrolled confounding, we present the E-value for the all-cause mortality effect estimate [27]. The E-value is “the minimum strength of association, on the risk ratio scale, that an unmeasured confounder would need to have with both the treatment and the outcome to fully explain away a specific treatment-outcome association, conditional on the measured covariates.” [27]

For CCR participants, we also examined factors collected at intake that could increase hazards of mortality. The model included the variable of interest (eg, insurance status) and adjusted for age, gender, transmission risk, race, treatment era, and baseline CD4 and VS.

Analyses were conducted in SAS V9.4.

## RESULTS

**Figure 1.**
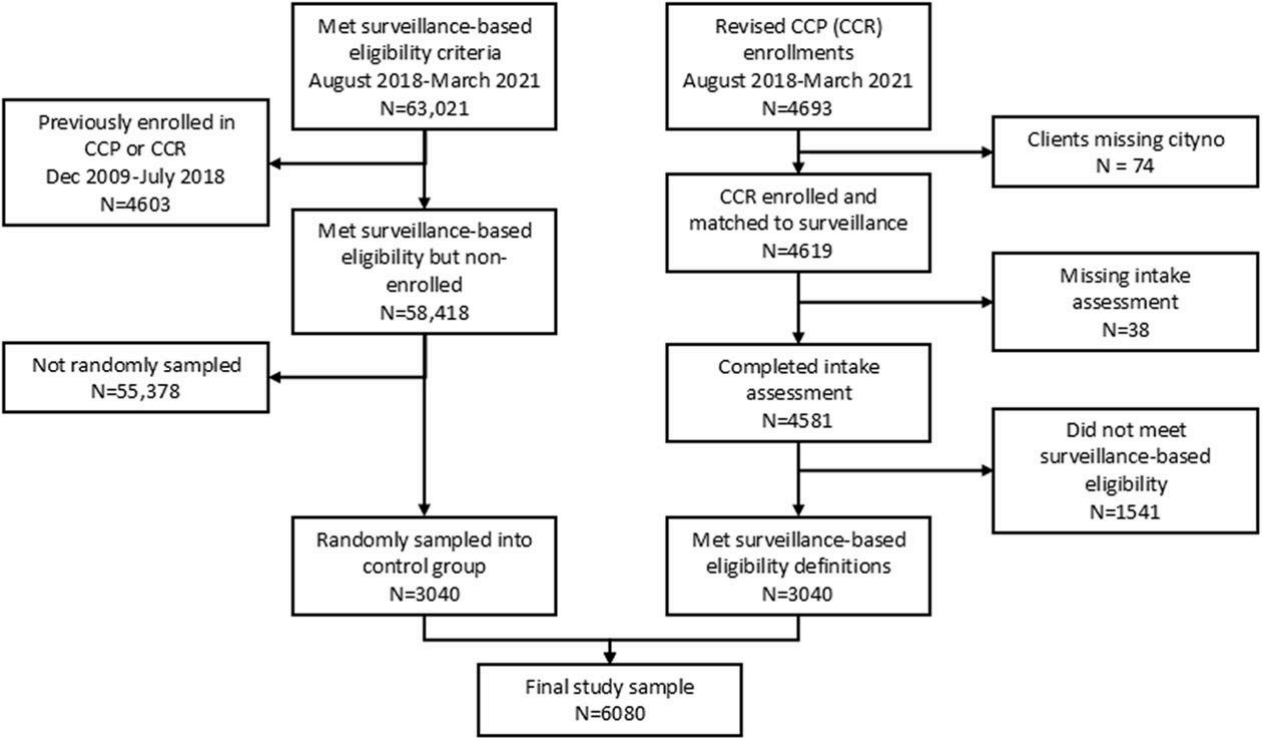
Participant flow diagram.

From 1 August 2018, to 31 March 2021, there were 4693 CCR enrollees, of whom 4581 (98%) completed a program intake assessment and 3040 (65%) met the surveillance-based eligibility criteria (Figure 1). There were 63 021 case records in the Registry that met the surveillance-based CCR eligibility criteria for at least 1 month during the study period. Of these, 58 418 (93%) were not enrolled in the CCR or the original CCP from December 2009 to March 2021. From this group, 3040 individuals were randomly selected and assigned a pseudo-enrollment date.

At enrollment, 43% of CCR participants were aged 50 years or older, 27% had a CD4 count <200 cells/µL, and 19% were diagnosed <1996 (Table 1). In the eligible non-CCR group, 41% were aged 50 years or older, 22% had a CD4 count <200 cells/µL, and 16% were diagnosed <1996. The IPTWs had a mean of 1 and standard deviation between 0.4 and 1. The CCR and non-CCR groups were balanced on all characteristics after applying the IPTWs.

**Table 1.** Characteristics of Non-CCR and CCR Participants—August 2018 to March 2021, New York City

	Total	
	Non-CCR	CCR
Total	3040 (100.00)	3040 (100.00)
Age		
0–17	14 (0.46)	3 (0.10)
18–29	518 (17.04)	511 (16.81)
30–49	1262 (41.51)	1216 (40.00)
50–64	1010 (33.22)	1104 (36.32)
65+	236 (7.76)	206 (6.78)
Age 50+		
No	1794 (59.01)	1730 (56.91)
Yes	1246 (40.99)	1310 (43.09)
Gender^a^		
Cisgender female	811 (26.68)	945 (31.09)
Cisgender male	2149 (70.69)	1984 (65.26)
TGNCNB	80 (2.63)	111 (3.65)
Race/Ethnicity		
API and other	85 (2.80)	74 (2.43)
Black	1579 (51.94)	1613 (53.06)
Hispanic	1005 (33.06)	1228 (40.39)
White	371 (12.20)	125 (4.11)
Transmission risk		
Heterosexual	659 (21.68)	731 (24.05)
IDU and MSM-IDU	424 (13.95)	583 (19.18)
MSM	1255 (41.28)	1027 (33.78)
Other^b^	163 (5.36)	212 (6.97)
Unknown	539 (17.73)	487 (16.02)
HIV prevalence/poverty^c^		
High poverty/high HIV prevalence	1014 (33.36)	1239 (40.76)
High poverty/low HIV prevalence	505 (16.61)	622 (20.46)
Low poverty/high HIV prevalence	283 (9.31)	197 (6.48)
Low poverty/low HIV prevalence	972 (31.97)	939 (30.89)
Unknown	266 (8.75)	43 (1.41)
Baseline CD4^d^		
<200	650 (21.38)	804 (26.45)
200–349	465 (15.30)	546 (17.96)
350–499	454 (14.93)	395 (12.99)
500+	1037 (34.11)	777 (25.56)
Unknown	434 (14.28)	518 (17.04)
Baseline VL^d^		
<200	1187 (39.05)	848 (27.89)
200+	1441 (47.40)	1705 (56.09)
Unknown	412 (13.55)	487 (16.02)
Treatment era based on diagnosis year		
Prior cART (<1996)	477 (15.69)	588 (19.34)
cART era (1996–2005)	949 (31.22)	976 (32.11)
Single-tablet era (2006+)	1614 (53.09)	1476 (48.55)
Treatment status^e^		
Consistently suppressed	279 (9.18)	279 (9.18)
Inconsistently suppressed	1108 (36.45)	1108 (36.45)
Lacking evidence of VS	1179 (38.78)	1179 (38.78)
Newly diagnosed	474 (15.59)	474 (15.59)
Insured^f^		
Uninsured	…	183 (6.02)
Insured	…	2705 (88.98)
Missing	…	152 (5.00)
Stably housed^f^		
No	…	681 (22.40)
Yes	…	2271 (74.70)
Missing	…	88 (2.89)
Recent drug use^f^		
No	…	2023 (66.55)
Yes	…	670 (22.04)
Missing	…	347 (11.41)
Depression^f^		
No	…	1894 (62.30)
Yes	…	1146 (37.70)
Psychosis^f^		
No	…	2574 (84.67)
Yes	…	466 (15.33)
PTSD^f^		
No	…	2835 (93.26)
Yes	…	205 (6.74)
level^f^		
High school or lower	…	2019 (66.41)
>High school	…	924 (30.39)
Missing	…	97 (3.19)

Abbreviations: API, Asian, Pacific Islander; cART, combination antiretroviral treatment; CCR, revised Care Coordination Program; IDU, injection drug use; MSM, men who have sex with men; TGNCNB, transgender, gender nonconforming or nonbinary; VL, viral load; >, viral suppression.

^a^Fewer than 5 participants were transgender men across both groups.

^b^Includes people with perinatal infection and transgender people with sexual contact.

^c^Residential ZIP code-level HIV prevalence and poverty were assigned based on each participant's most recent available ZIP code of residence in the Registry and classified as high (prevalence greater than median in the year prior to enrollment/pseudo-enrollment) or low (prevalence below median in the year prior to enrollment/pseudo-enrollment).

^d^In the year prior to enrollment/pseudo-enrollment.

^e^Baseline treatment status definitions: lacking evidence of VS: no VL results <=200 copies/mL in the year prior to enrollment/pseudo-enrollment; newly diagnosed: diagnosed with HIV in the year prior to enrollment/pseudo-enrollment; inconsistently suppressed: at least 1 VL result <=200 copies/mL and 1 VL result >200 copies/mL in the year prior to enrollment/pseudo-enrollment; consistently suppressed: all VL results <=200 copies/mL in the year prior to enrollment/pseudo-enrollment.

^f^Captured in the programmatic database and data not available for non-CCR comparison population.

### All-cause and HIV-related Mortality

Among CCR participants, the all-cause and HIV-related mortality rates were 3.31 per 100 person years (PY) and 0.70 per 100 PY, respectively. Among controls, the all-cause and HIV-related mortality rates were 3.69 and 1.17, respectively, per 100 PY. In adjusted analyses, the CCR participants had lower all-cause and HIV-related mortality relative to their counterparts receiving usual care, and these effects were consistent among PAWH and younger PWH, among those lacking evidence of VS in the year before enrollment/pseudo-enrollment, among those diagnosed in later years (2006+), and among those with baseline CD4 counts <200 and 200–349 cells/µL (Table 2). Among PWH diagnosed from 1996 to 2005 and among PWH who were inconsistently suppressed in the preenrollment/pseudo-enrollment year, CCR participants had lower HIV-related but not all-cause mortality.

**Table 2.** All-cause and HIV-related Mortality Rates by Baseline Treatment Status Group, Age at Enrollment, Diagnosis Year, and Baseline CD4 Count Among PWH Enrolled in the CCR Relative to Usual Care (Non-CCR)—New York City, 01 August 2018 to 31 March 2022

	Total Persons				All-cause Mortality						HIV-related Mortality					
	Non-CCR		CCR		Non-CCR		CCR		…	…	Non-CCR		CCR		…	…
	Persons	PY	Persons	PY	Deaths	Crude Rate (Per 100 PY)	Deaths	Crude Rate (Per 100 PY)	Crude HR	Adj HR	Deaths	Crude Rate (Per 100 PY)	Deaths	Crude Rate (Per 100 PY)	Crude HR	Adj HR
Total	3040	8390	3040	8580	310	3.69	284	3.31	0.90 (0.76, 1.05)	**0.71 (0.60, 0.84)**	98	1.17	60	0.7	**0.60 (0.43, 0.83)**	**0.43 (0.31, 0.60)**
Treatment status	…	…	…	…	…	…	…	…	…	…	…	…	…	…	…	…
Newly diagnosed	474	1399	474	1403	8	0.57	9	0.64	1.12 (0.43, 2.90)	0.73 (0.28, 1.91)	3	0.21	2	0.14	0.66 (0.11, 3.98)	0.34 (0.05, 2.09)
Consistently suppressed	279	787	279	771	28	3.56	39	5.06	1.41 (0.87, 2.29)	1.20 (0.75, 1.93)	8	1.02	5	0.65	0.63 (0.21, 1.94)	0.45 (0.15, 1.39)
Lacking evidence of VS	1179	3085	1179	3302	178	5.77	119	3.60	**0.63 (0.50, 0.79)**	**0.55 (0.43, 0.69)**	64	2.07	33	1	**0.48 (0.32, 0.74)**	**0.39 (0.25, 0.60)**
Inconsistently suppressed	1108	3120	1108	3104	96	3.08	117	3.77	1.22 (0.93, 1.60)	0.86 (0.66, 1.13)	23	0.74	20	0.64	0.87 (0.48, 1.59)	**0.54 (0.29, 1.00)**
Age 50+ at enrollment	…	…	…	…	…	…	…	…	…	…	…	…	…	…	…	…
No	1794	5130	1730	5013	100	1.95	95	1.90	0.97 (0.73, 1.29)	**0.75 (0.56, 0.99)**	36	0.7	27	0.54	0.77 (0.47, 1.26)	**0.47 (0.28, 0.78)**
Yes	1246	3260	1310	3567	210	6.44	189	5.30	**0.82 (0.68, 1.00)**	**0.70 (0.57, 0.86)**	62	1.9	33	0.93	**0.49 (0.32, 0.74)**	**0.41 (0.26, 0.63)**
Diagnosis year	…	…	…	…	…	…	…	…	…	…	…	…	…	…	…	…
<1996	477	1238	588	1574	90	7.27	104	6.61	0.91 (0.68, 1.20)	0.82 (0.62, 1.09)	27	2.18	27	1.72	0.79 (0.46, 1.34)	0.75 (0.43, 1.28)
1996–2005	949	2563	976	2704	117	4.56	116	4.29	0.94 (0.73, 1.21)	0.80 (0.62, 1.04)	32	1.25	21	0.78	0.62 (0.36, 1.08)	**0.47 (0.27, 0.84)**
2006+	1614	4589	1476	4303	103	2.24	64	1.49	**0.66 (0.49, 0.91)**	**0.52 (0.38, 0.71)**	39	0.85	12	0.28	**0.33 (0.17, 0.63)**	**0.20 (0.10, 0.40)**
Baseline CD4	…	…	…	…	…	…	…	…	…	…	…	…	…	…	…	…
<200	650	1632	804	2141	134	8.21	144	6.73	0.82 (0.65, 1.04)	**0.71 (0.56, 0.90)**	48	2.94	42	1.96	0.67 (0.44, 1.01)	**0.55 (0.36, 0.83)**
200–349	465	1273	546	1541	54	4.24	46	2.99	0.70 (0.48, 1.04)	**0.57 (0.38, 0.85)**	16	1.26	6	0.39	**0.31 (0.12, 0.79)**	**0.24 (0.09, 0.68)**
350–499	454	1287	395	1135	36	2.80	31	2.73	0.98 (0.60, 1.58)	0.78 (0.48, 1.26)	10	0.78	4	0.35	0.45 (0.14, 1.45)	0.41 (0.13, 1.30)
500+	1037	3035	777	2239	35	1.15	47	2.10	**1.81 (1.17, 2.81)**	1.48 (0.95, 2.30)	9	0.30	7	0.31	1.05 (0.39, 2.82)	0.59 (0.21, 1.67)
Unknown	434	1163	518	1525	51	4.39	16	1.05	**0.24 (0.14, 0.42)**	**0.27 (0.15, 0.49)**	15	1.29	1	0.07	**0.05 (0.01, 0.39)**	**0.05 (0.01, 0.44)**

Bold text indicates confidence intervals do not contain the null value of 1. The 4 treatment status groups were defined in terms of diagnosis or viral suppression in the 12 m prior to enrollment/pseudo-enrollment: (1) newly diagnosed, (2) consistently suppressed (≥2 VLs ≥90 days apart and all VLs ≤200 copies/mL), (3) lacking evidence of VS (all VLs reported >200 copies/mL or lacking any VL report), or (4) inconsistently suppressed (≥1 VL ≤200 copies/mL, but not all VLs ≤200 copies/mL). Diagnosis years were categorized in relation to HIV treatment eras with <1996 representing diagnoses prior to combination antiretroviral treatment (cART), 1996–2005 representing the cART era, and diagnoses since 2005 representing the single-tablet era. We classified as “HIV-related” deaths for which the underlying cause recorded in the death certificate was coded with one of the ICD-10 codes representing HIV disease, B20-B24, 098.7, or R75.18. These codes correspond with opportunistic infections (eg, pneumocystis carinii pneumonia and cytomegaloviral infection), malignant neoplasms (Kaposi sarcoma and Burkitt lymphoma), other specific diseases or infections associated with HIV infection, and unspecified conditions consistent with HIV disease or AIDS.

Abbreviations: Adj, adjusted; CCR, revised Care Coordination Program; HR, hazards ratio; non-CCR, persons eligible yet not enrolled in the CCR; PWH, persons with HIV; PY, person years; VS, viral suppression.

### Non-HIV-related Mortality

Non-HIV-related mortality was lower among CCR participants than controls, although the difference was not statistically significant (aHR: 0.85 [0.70–1.03]). The 2 most common causes of non-HIV-related mortality among CCR participants and controls were major cardiovascular disease and malignant neoplasms. Risk of death from major cardiovascular disease was significantly lower among CCR participants (aHR: 0.64 [0.42–0.98]) and risk of death from malignant neoplasms was nonsignificantly lower (aHR: 0.63 [0.39–1.04]) (Supplementary Table 4).

### Assessment of Unmeasured Confounding

With an observed hazards ratio (HR) of 0.71 for all-cause mortality, an unmeasured confounder that was associated with both all-cause mortality (outcome) and enrollment into CCR (exposure) by a risk ratio of 2.2-fold each, above and beyond the measured confounders, could explain away the estimate, but weaker joint confounder associations could not explain away the estimate. For the confidence interval to include the null, an unmeasured confounder that was associated with the outcome and exposure by a risk ratio of 1.7-fold each could do so, but weaker joint associations could not.

### Factors Associated With All-cause and HIV-related Mortality

All-cause mortality risk was higher among CCR participants with unstable (vs stable) housing (aHR: 1.32 [1.01–1.73]) and among CCR participants with PTSD (aHR: 1.51 [1.01–2.24]) (Table 3). Insurance status, recent drug use, depression/anxiety, psychosis, and education level were not significantly associated with all-cause mortality. No variables were significantly associated with HIV-related mortality.

**Table 3.** Baseline Characteristics Associated With All-cause and HIV-related Mortality Among CCR Enrollees

	Total Persons		All-cause Mortality				HIV-related Mortality			
	N	Sum PY	Deaths	Crude Rate (Per 100 PY)	Crude HR	Adjusted HR	Deaths	Crude Rate (Per 100 PY)	Crude HR	Adjusted HR
…	3040	8580	284	3.31	…	…	60	0.70	…	…
Housing status	…	…	…	…	…	…	…	…	…	…
Unstable	681	1896	82	4.32	1.43 (1.11, 1.85)	1.32 (1.01, 1.73)	12	0.63	0.90 (0.48, 1.71)	0.77 (0.40, 1.49)
Stable	2271	6437	194	3.01	Ref	Ref	45	0.70	Ref	Ref
Unknown	88	248	8	3.23	1.07 (0.53, 2.17)	1.32 (0.65, 2.70)	3	1.21	1.74 (0.54, 5.59)	2.85 (0.86, 9.40)
Recent drug use	…	…	…	…	…	…	…	…	…	…
No	2023	5713	185	3.24	…	…	38	0.67	…	…
Yes	670	1874	71	3.79	1.17 (0.89, 1.54)	1.02 (0.77, 1.34)	15	0.80	1.20 (0.66, 2.19)	1.06 (0.57, 1.96)
Unknown	347	994	28	2.82	0.87 (0.58, 1.29)	0.94 (0.63, 1.40)	7	0.70	1.06 (0.47, 2.37)	1.23 (0.54, 2.77)
Depression/anxiety	…	…	…	…	…	…	…	…	…	…
No	1894	5364	164	3.06	…	…	33	0.62	…	…
Yes	1146	3216	120	3.73	1.22 (0.96, 1.54)	0.95 (0.74, 1.21)	27	0.84	1.36 (0.82, 2.27)	1.36 (0.82, 2.27)
Psychosis	…	…	…	…	…	…	…	…	…	…
No	2574	7291	233	3.20	…	…	53	0.73	…	…
Yes	466	1290	51	3.95	1.24 (0.91, 1.67)	1.20 (0.83, 1.63)	7	0.54	0.75 (0.34, 1.64)	0.68 (0.31, 1.51)
PTSD	…	…	…	…	…	…	…	…	…	…
No	2835	8019	256	3.19	…	…	54	0.67	…	…
Yes	205	561	28	4.99	1.56 (1.06, 2.31)	1.51 (1.01, 2.24)	6	1.07	1.59 (0.68, 3.69)	1.39 (0.59, 3.27)
Education	…	…	…	…	…	…	…	…	…	…
>High school/GED	924	2655	68	2.56	…	…	17	0.64	…	…
High school/GED or lower	2019	5652	207	3.66	1.43 (1.09, 1.88)	1.33(0.97, 1.78)	40	0.71	1.10 (0.63, 1.95)	0.85 (0.47, 1.52)
Unknown	97	273	9	3.30	1.29 (0.64, 2.58)	1.18 (0.59, 2.53)	3	1.10	1.72 (0.50, 5.87)	2.31 (0.66, 8.12)

Adjusted model includes age, gender, transmission risk, race, treatment era, baseline CD4, and baseline VL.

Abbreviations: CCR, revised Care Coordination Program; GED, general educational development; HR, hazards ratio; PTSD, posttraumatic stress disorder; PY, person years; VL, viral load.

## DISCUSSION

Relative to usual care, the CCR may reduce all-cause mortality among populations at risk for poor outcomes, including PAWH and younger PWH, PWH lacking evidence of VS, and PWH with CD4 <350 cells/µL. All-cause mortality reductions among CCR participants were primarily driven by decreases in HIV-related mortality and accompanied by some decreases in non-HIV-related causes of mortality. Comparative effectiveness studies do not usually focus on mortality, as interventions target gaps further upstream in the care continuum that would lead to mortality. Care coordination has emerged as an effective intervention for retention in care and for VS and durable VS [12, 13, 16, 22, 28], and our data suggest that the CCR can also be an effective intervention for reducing HIV-related mortality and mortality from some non-HIV-related causes. In the case of HIV-related mortality, the mechanism is likely through improved care retention and sustained VS. The finding of an effect on some non-HIV-related causes is notable, suggesting the CCR may be supporting timely identification and/or improved management of other conditions through effective coordination of care with non-HIV specialists.

Identifying evidence-based strategies for PAWH is essential for individual and public health, given that up to three-quarters of PWH in the United States will be >50 years of age by 2040. As of October 2025, the Compendium of Evidence-Based Interventions and Best Practices for HIV Prevention did not include any intervention with an evidence-based designation for PAWH [28, 29]. Programs such as the SF Golden Compass offer models of aging-focused care; however, comparative effectiveness data are unavailable [29, 30]. Cumulative HIV viremia has been associated with increased risk of myocardial infarction, stroke and cardiovascular disease in some cohort studies [31–34]. Thus, achieving and maintaining VS is an important factor in preventing and treating comorbidities associated with HIV and may be another mechanism through which care coordination addresses some non-HIV-related causes of mortality [35, 36]. Relative to the general population, PWH have a greater burden of chronic conditions, such as cancers, metabolic disorders [2, 37], and mental health disorders [38, 39] and a greater prevalence of risk factors for chronic conditions, such as smoking and substance use [40, 41]. Further increasing life expectancy, especially for PAWH, will require treating and preventing chronic conditions other than HIV [42]. Models, such as care coordination, that integrate HIV, primary, and specialty care, along with support for psychosocial and basic needs, may yield broader health and survival gains beyond the HIV continuum.

Among enrollees, assessing for PTSD or experience of unstable housing could help to identify those at higher risk for mortality. Study results highlight the importance of stratified care and treatment planning at the time of enrollment. Future research should explore how addressing barriers such as housing instability may reduce risk of mortality. We did not observe significant risk factors for HIV-related causes of death, but this may be due to small samples and reduced power.

Our study's strengths include the application of a target trial emulation, which enhances validity by ensuring alignment of eligibility and follow-up across CCR and non-CCR PWH, thus avoiding common design flaws that contribute to selection bias [21]. Another strength was the use of population-based outcome data, ensuring we had local and national mortality and cause of death captured among CCR and non-CCR PWH. However, individuals were not randomized to the CCR, and unmeasured confounding remains a concern. We were unable to control for mortality risk factors, such as smoking or substance use, which may differ between CCR and non-CCR-enrolled PWH. Such risk factors would need to be associated with enrollment into the program and the outcome by a risk ratio of 2.2-fold each to explain away the observed effects of the CCR. In addition, our ability to detect small to moderate effect sizes may be limited by the sample size of some smaller subgroups of interest. Finally, our results are based on an ITI framework, which would move effect estimates closer to the null value.

Relative to usual care, the CCR shows benefits for reducing all-cause mortality among populations at risk for poor outcomes, including PAWH, younger PWH, and PWH lacking evidence of VS in the prior year. The impact on all-cause mortality was primarily driven by reductions in HIV-related mortality. These findings suggest that the CCR may be directly or indirectly addressing barriers to HIV treatment and adherence, as well as helping to treat and prevent other chronic conditions among PWH.

## Supplementary Material

ofag206_Supplementary_Data

## Supplementary Data

Supplementary materials are available at *Open Forum Infectious Diseases* online. Consisting of data provided by the authors to benefit the reader, the posted materials are not copyedited and are the sole responsibility of the authors, so questions or comments should be addressed to the corresponding author.
