# Supplementary material for: Impact of an HIV Care Coordination Program on All-cause, HIV-related, and Non-HIV-related Mortality in Younger and Older Adults With HIV in New York City, 2018–2022
Source: Open Forum Infect Dis. 2026 Apr 23;13(5):ofag206. doi: 10.1093/ofid/ofag206 (PMC13166153; doi:10.1093/ofid/ofag206)
Supplement: ofag206_Supplementary_Data [file ofag206_supplementary_data.docx]

| **Supplemental Table 1. Target trial protocol for comparing CCR enrollment with usual care** | | |
| --- | --- | --- |
| **Protocol Element** | **Target Trial Specification** | **Target Trial Emulation** |
| Eligibility Criteria | Individuals who met any of the following criteria:   - Newly diagnosed with HIV in the past 12 months - Virally unsuppressed at most recent known viral load test in the past 12 months - Out of care for at least 9 months - Currently living with hepatitis C and HIV - Currently pregnant and living with HIV - New to treatment/ART initiation - Undergoing change in treatment regimen - Previously diagnosed and at high risk of falling out of medical care or becoming unsuppressed   Baseline was defined as the first date when any of the above criteria are met | Surveillance-based definitions of eligibility criteria (see Supplemental Table 2)  Baseline was defined as the date of program enrollment OR pseudo-enrollment: a randomly assigned date within a window of time when at least one of the eligibility criteria were met |
| Treatment strategies | i) Enrollment in the revised care coordination program (CCR)  ii) Usual care | Same as target trial |
| Treatment assignment | Individuals were assigned a treatment strategy at baseline. Individuals and their providers were aware of the assigned treatment strategy | Individuals were classified into 1 of 2 groups according to the strategy that fit their data at baseline. We identified all clients enrolled in CCR and randomly sampled eligible controls using HIV registry data. We emulate randomization by adjusting for baseline confounders |
| Outcomes | All-cause mortality and HIV-related mortality | Same as target trial |
| Follow-up | Individuals contributed time from enrollment/pseudo-enrollment to the earlier of March 31, 2022, 36 months (1095 days) after enrollment/pseudo-enrollment, or death | Same as target trial, using assigned pseudo-enrollment date as start of follow-up for non-enrolled group |
| Causal contrasts | Intention-to-treat effect | Observational analogue of intention-to-treat effect |
| Statistical analysis | Application of inverse-probability weights to adjust for pre- and post-baseline prognostic factors associated with confounding and loss to follow-up | Same as target trial with adjustment for baseline confounders |

| **Supplemental Table 2 - Revised Care Coordination Program Registry-based eligibility criteria** | | | |
| --- | --- | --- | --- |
| **Program Eligibility Criteria** | **Registry-based Definition** | **Eligibility Window Start^1^** | **Eligibility Window End^2^** |
| Newly diagnosed with HIV in the past 12 months | Individuals diagnosed with HIV between 8/1/2017 and 3/31/2021 | Date of diagnosis | 12 months after diagnosis date |
| Virally unsuppressed at most recent known viral load test within the past 12 months | Individuals with a VL >= 200 copies/mL reported from 8/1/2017 to 3/31/2021 | Date of VL lab report with result >= 200 copies/mL | 12 months after the date of high VL lab report |
| Not consistently in care: Out of care for at least 9 months^3^ | Individuals without any CD4 or VL laboratory tests reported for any 9-month period (after diagnosis) from 11/1/2017 to 3/31/2021 | 9 months after a laboratory test | Laboratory test date following the 9-month or longer period without laboratory monitoring |
| Currently living with hepatitis C and HIV | Individuals with HIV and with an active HCV infection between 8/1/2017 and 3/31/2021 | Month of HCV diagnosis^4^ | HCV cure date^5^ |
| Currently pregnant and living with HIV | Individuals with HIV with a date of delivery from 8/1/2018 to 3/31/2021 | 9 months before the date of delivery | Date of delivery |
| New to treatment/ART initiation | First instance after HIV diagnosis of a lab report of a VL < 200 copies/mL OR 1^st^ log 1 decrease in VL | Month of 1^st^ VL < 200 copies/mL OR 1^st^ log 1 decrease in VL | 2 months after start of eligibility window |
| ^1^If eligibility window start date was before the start of the study period (i.e., 9/1/2017), then the eligibility window start date was set to 9/1/2017. | | | |
| ^2^If the eligibility window end date was after the end of the study period (i.e., 3/31/2021) OR if the individual died prior to the end of their eligibility window, then the eligibility window end date was set to the earlier of their death date or 3/31/2021. | | | |
| ^3^If an individual has a gap for more than 2 years, they werre considered out of jurisdiction and thus ineligible. | | | |
| ^4^First positive result on an HCV RNA test reported to the HCV registry between 8/1/2017 and 3/31/2021 | | | |
| ^5^Negative result on the most recent HCV RNA test following the most recent positive HCV RNA test between 8/1/2017 and 3/31/2021 | | | |

| **Supplemental Table 3: Variables included in IPTW models, by baseline treatment status** | |
| --- | --- |
| Baseline treatment status^1^ | Variables included |
| Lacking evidence of VS | Age at enrollment/pseudo-enrollment, gender, race/ethnicity, transmission risk category, baseline VL and CD4,^2^ year of HIV diagnosis, concurrent AIDS diagnosis,^3^ number of VL reports in the past year, ZIP code-level prevalence of HIV, ZIP code-level prevalence of poverty |
| Newly diagnosed with HIV | Age at enrollment/pseudo-enrollment, gender, race/ethnicity, transmission risk category, baseline VL and CD4,^2^ concurrent AIDS diagnosis,^3^ ZIP code-level prevalence of HIV, ZIP code-level prevalence of poverty |
| Inconsistently suppressed | Age at enrollment/pseudo-enrollment, gender, race/ethnicity, HIV transmission risk category, baseline VL and CD4,^2^ year of HIV diagnosis, concurrent AIDS diagnosis,^3^ number of VL reports in the past year, ZIP code-level prevalence of HIV, ZIP code-level prevalence of poverty |
| Consistently suppressed | Age at enrollment/pseudo-enrollment, gender, race/ethnicity, transmission risk category, year of HIV diagnosis, concurrent AIDS diagnosis,^3^ ZIP code-level prevalence of HIV, ZIP code-level prevalence of poverty |
| VL: viral load, VS: viral suppression  ^1^Baseline treatment status definitions: Lacking evidence of VS: no VL results <200 copies/mL in the year prior to enrollment/pseudo-enrollment; newly diagnosed: diagnosed with HIV in the year prior to enrollment/pseudo-enrollment; inconsistently suppressed: at least 1 VL result <200 copies/mL AND 1 VL result > 200 copies/mL in the year prior to enrollment/pseudo-enrollment; consistently suppressed: all VL results <200 copies/mL in the year prior to enrollment/pseudo-enrollment  ^2^Most recent VL or CD4 in the year prior to enrollment/pseudo-enrollment  ^3^AIDS diagnosis within 1 year of HIV diagnosis | |

**Supplemental Table 4. All-Cause, HIV-Related, Non-HIV-Related, Major Cardiovascular Disease and Malignant Neoplasms Mortality Rates among PWH Enrolled in the CCR Relative to Usual Care (non-CCR) – New York City, August 01, 2018-March 31, 2022**

|  | Non-CCR Person Years = 8390 | | | CCR Person Years = 8580 | | |  |  |
| --- | --- | --- | --- | --- | --- | --- | --- | --- |
|  | Deaths | % | Crude Rate Per 100 PY | Deaths | % | Crude Rate Per 100 PY | Crude HR | Adj. HR |
| **All-Cause** | 310 | 100 | 3.69 | 284 | 100 | 3.31 | **0.90 (0.76, 1.05)** | **0.71 (0.60, 0.84)** |
| **HIV Related** | 98 | 31.61 | 1.17 | 60 | 21.13 | 0.70 | **0.60 (0.43, 0.83)** | **0.43 (0.31, 0.60)** |
| **Non-HIV Related** | 212 | 68.39 | 2.53 | 224 | 78.87 | 2.61 | 1.03 (0.86, 1.24) | 0.85 (0.70, 1.03) |
| Age 50+ at Enrollment |  |  |  |  |  |  |  |  |
| No | 64 | 20.65 | 1.24 | 68 | 23.94 | 1.36 | 1.09 (0.77, 1.53) | 0.93 (0.66, 1.31) |
| Yes | 148 | 47.74 | 4.54 | 156 | 54.92 | 4.37 | 0.96 (0.77, 1.20) | 0.82 (0.66, 1.04) |
| Major Cardiovascular Disease | 49 | 15.81 | 0.58 | 40 | 14.08 | 0.47 | 0.80 (0.52, 1.21) | **0.64 (0.42, 0.98)** |
| Malignant Neoplasms | 36 | 11.61 | 0.43 | 30 | 10.56 | 0.35 | 0.81 (0.50, 1.32) | 0.63 (0.39, 1.04) |
| Adj: Adjusted; CCR: Revised Care Coordination program, HR: hazards ratio, Non-CCR: persons eligible yet not enrolled in the CCR, PWH: persons with HIV, PY: Person Years | | | | | | | | |
| We classified as “HIV-related” deaths for which the underlying cause recorded in the death certificate was coded with one of the ICD-10 codes representing HIV disease, B20-B24, 098.7, or R75.[18](https://paperpile.com/c/VNnrb0/tKDH). These codes correspond with opportunistic infections (e.g., pneumocystis carinii pneumonia and cytomegaloviral infection), malignant neoplasms (Kaposi sarcoma and Burkitt lymphoma), other specific diseases or infections associated with HIV infection, and unspecified conditions consistent with HIV disease or AIDS. | | | | | | | | |
